# Empyema—II

**Published:** 1908-01-04

**Authors:** 


### Points in Surgery.

### EMPYEMA.—II.

If pus is found with the exploring syringe it is imperative that free drainage should be given to it by operation without further delay.

The patient should be anæsthetised, preferably with chloroform. Ether, although theoretically a safer anæsthetic for general purposes than chloroform, is not so in this case, because it causes much mucous secretion in the bronchi, which may further impede the patient's respiration, already embarrassed as it is by the pressure of the fluid in his thorax. It is true that the induction of anæsthesia with pure chloroform is sometimes accompanied by struggling; but the truth is that this is due more to lack of skill on the part of the anæsthetist than on the particular form of anæsthetic employed; and if chloroform is properly given the patient should go under quite quietly. If the anæsthetist has any doubt on this point a mixture of two parts of chloroform and three parts of ether may be used on an open mask to induce anæsthesia, and pure chloroform may be used subsequently. The anæsthesia need be quite light, it being necessary to give only quite a small amount after the skin incision has been made. After this the patient should be allowed to come round a little, so that he begins to cough when the parietal pleura is opened, and thus assists in getting rid of the pus in the pleural cavity.

### The Incision.

Some difference of opinion exists as to the best spot for making the incision in operating on an empyema. Of course if the pus is localised the incision must be made over the affected area *secundum artem*. But if this is not the case, the thorax should be opened at the spot which gives the best drainage subsequently, whether the patient is sitting up or lying down. If one considers the anatomy of the thoracic wall, it is obvious that the most dependent part of the thorax where pus is likely to collect is the deep para-vertebral groove on either side of the spinal column. This is most conveniently opened by an incision along the eighth or ninth rib in the posterior axillary line. To do this the patient should lie on his back, well over to the edge of the table, so that by stooping or sitting down the surgeon can make his incision from below. The arm of the affected side should be strongly adducted, so that the hand is on the opposite shoulder. It may be necessary to tilt the patient *slightly* towards the sound side; but he should never under any circumstances lie completely on the sound side, as, for instance, in the nephrectomy position, because the lung of the affected side is to all intents and purposes useless as a respiratory apparatus, and in this position his only sound lung is hampered in its work, and if it is much embarrassed death may result.

When the patient is in position and all is ready, an incision is made about three inches in length over the eighth or ninth rib, in the posterior axillary line. The incision should go straight down to the bone. The tissues on each side are retracted to expose the rib, and an incision is made in the periosteum, which is then raised from the rib with an elevator. A portion of rib, about an inch or an inch and a half, is removed with bone forceps, and the point of the forefinger introduced to examine the state of affairs. If the ninth rib has been chosen, it must be remembered that the dome of the diaphragm rises into the thorax almost vertically up to this level, and this muscle may easily be opened under the mistaken impression that it is the parietal pleura. The writer has seen this done on more than one occasion. The danger of doing so is the risk of setting up peritonitis. The parietal pleura can be found by pushing the examining finger upwards. If there is any difficulty in exposing it, a portion of the eighth should be removed as well, so as to get a good exposure. The pleura should now be opened with a scalpel, and the pus immediately escapes. If the patient is not deeply anæsthetised he begins to cough at this moment and the expansion of lung so caused helps to drive out the pus. The opening in the pleura should be quite a small one, so that the pus can come out gradually at first. It can be enlarged afterwards with sinus forceps if necessary. The danger of making a large opening at once is that the sudden relief of tension may lead to acute cardiac dilatation, and the patient may even die on the operating table.

### Exploring the Cavity.

The condition of the pleura should now be examined with the finger. In this way the extent of the pus containing cavity may be made out and the flocculent masses which are nearly always to be found, especially in a pneumococcal case, can be brought to the surface. Irrigation of the cavity is a dangerous practice, which has been known to cause the death of the patient: it should, therefore, be avoided. When the spontaneous ejection of pus has ceased a tube should be inserted. There is no better form of apparatus than the flanged india-rubber tube known as an empyema tube. It is kept in position by tapes which are attached to the flange, and are fastened round the thorax. It has been said that it tends to get kinked by the ribs, and the use of solid tubes made of celluloid, silver, and other materials has been advocated; but an ordinary empyema tube answers the purpose perfectly well, if it is examined daily. Special care should be taken to see that the tube does not slip into the thorax; this has been known to occur, and is a most troublesome complication to deal with.

In a pneumococcal case the discharge is fairly free for three or four days, and then tends to cease or become serous. When this happens the tube may be left out altogether, and the condition generally clears up completely in ten days or a fortnight. But if the pus is of a streptococcal nature or becomes secondarily infected at the operation or afterwards, it is probable that it will continue to drain away for weeks or even months, and the prognosis is not at all good. Many of these patients eventually succumb to amyloid disease.

